# Supplementary material for: Resolving the full spectrum of human genome variation using Linked-Reads
Source: Genome Res. 2019 Apr;29(4):635–45. doi: 10.1101/gr.234443.118 (PMC6442396; doi:10.1101/gr.234443.118)
Supplement: Supplemental Material [file supp_gr.234443.118_Supplemental_longranger-2.2.zip › longranger-2.2.tar.bz2!/lr2.2-public/lib/stan/cmdstan/stan/doc/api/html/io_2util_8hpp_source.html]

Stan: src/stan/io/util.hpp Source File


|  |  |
| --- | --- |
|  | Stan  2.5.0  probability, sampling & optimization |


- Main Page
- Related Pages
- Namespaces
- Classes
- Files

- File List
- File Members

All Classes Namespaces Files Functions Variables Typedefs Enumerations Enumerator Friends Macros Pages

- src
- stan
- io

util.hpp

Go to the documentation of this file.

1 #ifndef STAN\_\_IO\_\_UTIL\_HPP

2 #define STAN\_\_IO\_\_UTIL\_HPP

3

4 #include <string>

5 #include <ctime>

6

7 namespace stan {

8

9  namespace io {

10

18  std::string utc\_time\_string() {

19  // FIXME: use std::strftime

20

21  // original with asctime

22  // std::time\_t rawtime = time(0);

23  // std::tm \*time = gmtime(&rawtime);

24  // return std::string(asctime(time));

25

26  // new with strfitime

27  time\_t rawtime;

28  std::time(&rawtime);

29

30  char cbuf[80];

31  std::strftime(cbuf,80,"%a %b %d %Y %H:%M:%S",

32  std::localtime(&rawtime));

33

34  return std::string(cbuf);

35  }

36

37  }

38 }

39

40 #endif

stan::io::utc\_time\_string

std::string utc\_time\_string()

Return the current coordinated universal time (UTC) as a string.

**Definition:** util.hpp:18


---

[ Stan Home Page ]

*© 2011–2014,
Stan Development Team.*
